# A dietary approach to skin cancer prevention in solid organ transplant recipients

**DOI:** 10.3389/frtra.2026.1949295

**Published:** 2026-10-21

**Authors:** Kerry Yang, Melody Li, Taylor Skinner, Danlin Zeng, Gabriela Sosa, Jing Yi Han, Fatemeh Jafarian

**Affiliations:** 1 Cumming School of Medicine, University of Calgary, Calgary, AB, Canada; 2 Department of Dermatology and Skin Science, University of British Columbia, Vancouver, BC, Canada; 3 Discipline of Medicine, Memorial University of Newfoundland, St John’s, NL, Canada; 4 Faculty of Medicine, University of British Columbia, Vancouver, BC, Canada; 5 Division of Dermatology, Department of Medicine, University of Calgary, Calgary, AB, Canada

**Keywords:** chemoprevention, dermatology, diet, nutrition, skin cancer, solid-organ transplant, transplantation

## Abstract

Among all patient populations, solid organ transplant recipients are one of the most at-risk groups for cutaneous malignancies. While modern post-transplant treatment approaches have significantly improved life expectancy, these chronic immunosuppressive regimens often lead to the rapid proliferation of skin cancers such as squamous cell carcinoma, basal cell carcinoma, and melanoma. Despite advances in surveillance, photoprotection, and therapeutic strategies, there is still substantial morbidity and mortality due to cutaneous malignancies in transplant recipients. One potential low-risk approach to chemoprevention is through the diet. To gain a better understanding of skin cancer prevention using dietary compounds, a review of the current literature was conducted. By appraising the current evidence, there appears to be potential therapeutic benefit for a variety of different diets and nutritional compounds, including but not limited to the Mediterranean diet, omega-3 fatty acids, nicotinamide, and phytochemicals like polyphenols and carotenoids. Although significant knowledge gaps remain, dietary modification and supplementation could be a potentially effective approach to cutaneous chemoprevention among solid organ transplant recipients.

## 1 Introduction

Solid organ transplant recipients (SOTRs) are one of the highest-risk groups for cutaneous malignancies (1). While life-long immunosuppression has improved graft survival, significantly prolonging the life expectancy post-transplant, SOTRs face a 10-fold increased risk of basal cell carcinoma (BCC), a 65 to 250-fold increase in squamous cell carcinoma (SCC), and a threefold increased risk of melanoma compared to the general, immunocompetent population (2–4). Approximately half of all SOTRs will develop at least one skin cancer in their lifetime (5), with these cancers characterized by rapid growth, a higher likelihood of recurrence, and a significantly elevated risk of metastasis, leading to increased mortality rates (6–8). Despite advances in surveillance, photoprotection, and pharmacologic intervention with medications such as systemic retinoids (1, 8), there is still a substantial mortality burden from skin cancer post-transplant, especially due to non-melanoma skin cancers (NMSC) such as BCCs and SCCs. Thus, there continues to be a need for safe, minimally harmful adjunctive strategies.

One potential approach with a minimal risk for adverse effects is chemoprevention through the diet. Approximately 18% of global cancer burden is due to modifiable lifestyle factors, such as nutritional intake and adiposity (9). Dietary factors likely play an outsized role in SOTRs, especially given the immune-related effects of post-transplant regimens and the poor gastrointestinal tolerability of these drugs, which affects nutritional intake and absorption (10). Studies with immunocompetent and immunosuppressed cohorts have found a benefit from using various dietary compounds in reducing the incidence of malignancy (11, 12), and exploratory studies with SOTRs are starting to suggest the same (13). In order to better understand the current evidence, the primary goal of this perspective, with selective commentary is to explore potential dietary approaches to cutaneous chemoprevention for SOTRs based upon the current literature. A review of the current literature was conducted using two separate search strategies on PubMed, with the searches conducted between October 2025 to January 2026. A focus search was conducted using a transplant-related line [(kidney or liver or heart or lung or pancreas or solid organ) and transplant or solid organ recipient or “SOTR”], a skin cancer-related line (basal cell carcinoma or “BCC” or squamous cell carcinoma or “SCC” or non-melanoma skin cancer or “NMSC” or melanoma or skin cancer or actinic keratosis or “AK”), and a diet-related line (diet or food or nutrient or omega or fatty acid or nicotinamide or B3 or phytochemical or polyphenol or carotenoid or carotene). A broader search dropping the transplant-related line was utilized as well, with findings from both strategies extracted and synthesized.

## 2 Carcinogenesis and the skin

Skin carcinogenesis occurs through a convergence of oxidative, mutagenic, and immunologic processes, primarily driven by ultraviolet radiation (UVR). UV exposure induces reactive oxygen species (ROS) formation, which can damage cellular macromolecules such as DNA, proteins, and phospholipids, and influence gene expression through the modulation of multiple intracellular pathways (14–16), including the mitogen-activated protein kinase (MAPK) and nuclear factor kappa B (NF-*κ*B) signaling cascades (15, 17). This occurs when ROS overwhelm endogenous antioxidant defenses such as superoxide dismutase, glutathione peroxidase, and catalase, thereby promoting lipid peroxidation, DNA damage, mutations, and signaling alterations that contribute to UV-induced cutaneous carcinogenesis (18, 19). Deviations in these pathways can lead to hyperproliferation, angiogenesis, and the inhibition of apoptosis (15).

UVR can induce dipyrimidine photoproducts, such as cyclobutane pyrimidine dimers, within epidermal keratinocytes, distorting the helical structure of DNA (20). Although this is often manageable through apoptosis, cumulative UV exposure can overcome these apoptotic pathways, resulting in the hyperproliferation of aberrant keratinocytes (21).

Simultaneously, UVR exerts immunomodulatory effects within the localized skin environment. The apoptotic bodies left behind after UV radiation exposure can induce the production of the anti-inflammatory interleukin (IL)-10 while inhibiting the production of proinflammatory cytokines such as IL-12, tumour necrosis factor *α* (TNF- *α*), and interferon-*γ* (IFN- *γ*) (22–25). This limits the ability of the immune system to surveil and eradicate dysplastic skin cells, allowing cutaneous neoplasms to form (16, 23).

Solar radiation can also reduce epidermal Langerhans cells and impair their ability to migrate to regional lymph nodes (26). Langerhans cells and other dendritic immune actors within the dermis play a key role in priming antigen-specific and helper T cells, and depletion of these dermal cell populations disrupts local and systemic immune responses (27–29).

Although immunosuppressive maintenance regimens are essential for graft survival, their use also increases the rate of NMSC formation and growth (30, 31). Calcineurin inhibitors (CNIs), such as cyclosporine and tacrolimus, have been shown to decrease the DNA repair of cyclobutane pyrimidine dimers, inhibit apoptosis of mutated keratinocytes, and reduce the number of effector T-cells (32). Cyclosporine has also been demonstrated to upregulate vascular endothelial growth factor (VEGF) and transforming growth factor-*β* (TGF-*β*) within local tumour environments, promoting angiogenesis and leading to more aggressive NMSC phenotypes (32–36).

Azathioprine leads to the accumulation of 6-thioguanine in cellular DNA, amplifying the absorption of UVR and accelerating carcinogenesis (37, 38). While other agents such as mycophenolate mofetil and corticosteroids carry lower risks of malignancy, chronic immunosuppression remains a key driver of NMSC carcinogenesis (39–43). Thus, the need for safe chemopreventative strategies that limit potential harms continues to remain a top priority.

## 3 Dietary strategies for cutaneous chemoprevention

The convergence of oxidative stress, impaired DNA repair, and immunosuppression provides a biologically coherent framework for the beneficial role diet could play (12). Dietary compounds are involved in redox homeostasis, inflammatory signaling, and DNA repair, which are important for modulating UVR-related cellular damage (44–46). In SOTRs, where small perturbations can cause large changes, various dietary strategies have the potential to play an even greater role.

A commonly researched topic is understanding the impact of comprehensive dietary modification on cancer incidence and mortality (11, 47). Studied diets range from regimens focused on caloric restriction to isocaloric approaches, such as the low-fat and Mediterranean diets (11, 48, 49).

In a recent systematic review of randomized controlled trials (RCTs) that were one year or longer, Sauter et al. found low-calorie, low-fat, and the Mediterranean diet had statistically significant benefits on some cancer-related biomarkers and incidence for both primary and secondary prevention (11). Although most of these studies focused on breast cancer incidence among immunocompetent women, they noted that various dietary approaches were associated with up to 10% weight loss, which had previously demonstrated an independent benefit for cancer and cancer-related biomarkers (11). Depending on the immunosuppressive regimen, significant weight gain is a common side effect following transplantation, especially within the first year (50). For SOTRs with a baseline BMI greater than or equal to 25 kg/m^2^, weight loss was associated with improved survival outcomes (51, 52). Given the current knowledge gaps, understanding the relationship between dietary weight loss and cutaneous chemoprevention would be an interesting future research question.

There is limited or mixed evidence that high overall dietary fat intake could potentially increase the incidence of NMSC in humans, with some studies reporting that higher overall energy intake from fat correlated to an elevated risk of SCC among men (53–56). However, the results of clinical studies exploring the relationship between low-fat, isocaloric diets with actinic keratosis (AK) and NMSC risk among immunocompetent patients have been relatively mixed. Although one clinical intervention trial and some retrospective studies found a benefit for primary and secondary cutaneous malignancy prevention, epidemiological studies did not (12, 48, 53, 54, 57–59). Meanwhile, associations are emerging between adherence to the Mediterranean and Dietary Approaches to Stop Hypertension (DASH) diets, and a reduction in the incidence of BCCs (49, 60). This association has also been found in melanoma studies, especially among women and those with good vegetable intake (61, 62). However, these articles were predominantly dependent on patient-filled questionnaires, which are highly prone to recall bias. A higher dietary intake of inflammatory foods has also been associated with an increased risk of melanoma (63), providing further evidence that certain dietary patterns may reduce skin cancer incidence.

## 4 Omega-3 and omega-6 fatty acids

Some of the most interesting dietary compounds under investigation for cutaneous chemoprevention are polyunsaturated fatty acids, such as omega-3 fatty acids. Omega-3 fatty acids, like the essential short-chain *α*-linolenic acid, are found in plant sources such as flaxseed, chia, walnuts, and leafy green vegetables. Longer-chain omega-3s, such as eicosapentaenoic acid (EPA) and docosahexaenoic acid (DHA), are commonly found in fatty fish like tuna, trout, and salmon (45, 64, 65). While vegetable oils such as soybean, palm, and corn contain a mix of both, they contain more omega-6 compared to omega-3, making them the main dietary source of omega-6 fatty acids. Meats such as beef and poultry also have more omega-6s compared to omega-3 (66).

Omega-3 fatty acids generally form anti-inflammatory eicosanoids like resolvins and protectins, while omega-6s are often transformed into the pro-inflammatory eicosanoids (66, 67). For example, the omega-6 fatty acid arachidonic acid can be released in the skin after UV exposure, transforming into prostaglandin E-2 (PGE_2_) by COX enzymes, and behaving as a cutaneous tumour promoter (68–70). Meanwhile, omega-3 fatty acids like EPA and DHA compete for the same COX enzymes, reducing the formation of PGE_2_ and thus limiting tumour promotion (71).

In mice fed a diet high in fish oil, there was a decreased incidence and increased latency of UVB-induced skin cancer (68, 72). Meanwhile, epidemiological data found an association between higher intake of omega-3 fatty acids and reduced AK incidence, while a greater proportion of omega-3 to omega-6 fatty acids was associated with a reduction in SCC (53, 73). In patients undergoing chemotherapy for non-small cell lung cancer, omega-3s were shown to decrease erythema, reduce susceptibility to lipid peroxidation, and prevent UVR-induced immunosuppression (74–76).

Meanwhile regarding studies in transplant recipients, longitudinal data found that SOTRs who regularly consumed a high amount of omega-3 polyunsaturated fatty acids had an adjusted relative SCC risk reduction of 67% (77). Transplant recipients with a high intake of long-chain omega-3 fatty acids had an adjusted reduction of 60% in the risk of BCC as well, although interpretation is limited given the observational design (77). A pilot RCT with lung transplant participants found that taking an omega-3 supplement containing eicosapentaenoic acid and docosahexaenoic acid four times daily led to a decrease in the rate of skin cancer compared to the trial arm using an olive oil placebo, but did not meet statistical significance (13). Although the number of studies is relatively limited with small sample sizes, strong signals are emerging for cutaneous chemoprevention with omega-3 fatty acids in SOTRs.

## 5 Nicotinamide, niacin, and other vitamin B3 derivatives

Another micronutrient receiving increased attention is nicotinamide. Also called niacinamide, it is a form of niacin (vitamin B3) naturally found in many foods like meats, dairy products, legumes, beans, nuts, green leafy vegetables, cereals, coffee, and tea (78). As the precursor to nicotinamide adenine dinucleotide (NAD+), this B3 derivative plays a vital role in the generation of adenosine triphosphate (ATP) (79, 80).

In mouse studies, nicotinamide was shown to prevent UVR-induced photocarcinogenesis and photoimmunosuppression, with early studies involving human participants suggesting there may be reduced rates of AK and NMSC (78, 81). A 2015 phase III RCT known as the ONTRAC trial found that oral nicotinamide supplementation at 500 mg twice daily in healthy participants with two or more NMSCs in the previous 5 years led to a reduction in new SCCs, BCCs, and AKs by 23% compared to placebo at the 12-month mark (81). A separate RCT with immunocompetent patients also found a secondary prevention benefit at the four-month mark using the same dosing regimen (82).

A phase II pilot RCT conducted in 2016 with renal transplant patients found a non-significant relative reduction of 35% in NMSCs and 16% in AKs at the 6-month mark for patients using the 500 mg twice-daily supplementation schedule for secondary prevention (83). In the follow-up phase III RCT conducted with 158 SOTRs who had two or more keratinocyte cancers in proceeding five years, known as the ONTRANS trial, there was no statistically significant reduction in AKs or NMSCs at the one-year mark (84). However, the authors did note a trend towards fewer invasive SCCs, consistent with what was demonstrated in the ONTRAC trial. They stated the number of participants recruited and keratinocyte neoplasms observed was far less than what they calculated for in their pre-trial power analysis, and a large number of participants dropped out, meaning their study was significantly underpowered and likely has a high risk of type II error (84). Other studies found conflicting results. A single-center study involving 21 kidney transplant recipients with a history of NMSCs found no benefit with nicotinamide on the rate of AK development, while a separate study with a similar population did (85, 86). There were issues with these studies as well, including a lack of blinding, along with one study needing to drop the dose of nicotinamide supplementation from 1000 mg BID to 500 mg BID due to gastrointestinal side effects, which introduces confounding by indication.

Meanwhile, retrospective studies are consistent in finding a benefit. A retrospective cohort study with 47 SOTRs observed a significant reduction in SCC and SCC-in-situ, but not BCC (87). A large retrospective cohort study using the electronic health record data from the Veterans Affairs system in the United States found a significant reduction in the risk of cutaneous SCC associated with oral nicotinamide use in SOTRs who had 1 or 2 prior skin cancers (88). Within their overall study population, they found the largest benefit occurred when oral nicotinamide was initiated shortly after the first skin cancer developed, with a gradual attenuation of this protective effect when supplementation was started following each subsequent skin cancer (88). In a retrospective cohort analysis, the authors found that adults with at least one prescription for niacinamide with a mean follow-up time of approximately 4 years had a decrease in BCC, SCC, and melanoma incidence compared to age-matched controls (89). However, these retrospective designs limit interpretability and external validity.

Given the current data, there is likely minimal benefit in using nicotinamide for SOTRs with a significant history of NMSC. However, nicotinamide might be a viable strategy for primary and early secondary chemoprevention of cutaneous SCCs. Although data on AKs and BCCs is more mixed, all studies found nicotinamide to be quite safe and well-tolerated. Given the delicate medical status of SOTRs, increasing dietary vitamin B3 intake or using nicotinamide supplementation may be a potential safe and affordable approach to cutaneous primary chemoprevention in transplant recipients.

## 6 Phytochemicals, polyphenols, and carotenoids

The term phytochemical refers to a diverse range of bioactive compounds synthesized by plants that are gaining significant attention in the scientific community for their potential therapeutic effects on health (90–93). There have been over 10 000 different types reported, including polyphenols, carotenoids, curcuminoids, and furocoumarins (94, 95).

The proposed biological activity of dietary phytochemicals is largely attributed to their antioxidant and cytoprotective properties, including the ability to scavenge ROS and nitrogen species through electron donation and modulation of endogenous defenses through nuclear factor erythroid 2–related factor 2 (Nrf2) (96, 97). They can also block the UV-induced activation of the MAPK and NF-*κ*B signaling pathways, inhibiting the transcription of proliferative genes and inhibiting pathways that induce angiogenesis, the epithelial-mesenchymal transition, invasion, and dissemination (97–100).

Polyphenols are compounds characterized as having at least two phenyl rings and one or more hydroxyl substituents, with the main dietary sources being fruit juice, wine, tea, coffee, and beer (101, 102). Over 8000 polyphenol structural variants have been described, subdivided into groups based on the number of phenolic rings and linked structural elements. These include flavonoids, stilbenes, tannins, lignans, and phenolic acids (102, 103).

Polyphenols were observed to inhibit UV-induced tyrosine phosphorylation of epidermal growth factor receptor and early response genes and proteins, such as *c-fos*, *c-jun*, and *p53* (104). This prevents their activation, inhibiting cellular proliferation, inflammation, and DNA damage, and reducing the risk of photoaging and skin cancer (98, 104–106).

In human studies, short-term oral ingestion of polyphenol-rich red wine resulted in a modest increase in minimal erythema dose (MED) in healthy volunteers (107). Topical use of white and green tea extracts has been associated with reductions in UV-induced erythema, edema, and DNA damage (36, 103, 105, 108, 109), but no benefit was demonstrated for MED in an RCT using ingested green tea tablets (110). However, there does seem to be an association between regular black tea consumption and reduced SCC and BCC risk, with higher total caffeine intake, including caffeinated tea, also having a negative association with BCC risk (55, 111).

Carotenoids are red, orange, and yellow tetraterpenoid pigments that protect plants against excessive sunlight, primarily obtained through the diet, with carrots, tomatoes, and leafy green vegetables being key sources (112, 113). Carotenoids are divided into carotenes and xanthophylls (114). Carotenes, such as *β*-carotene and lycopene, are pure hydrocarbons, while xanthophylls, such as lutein and zeaxanthin, contain oxygenated functional groups (114). Chemically, carotenoids are characterized by a conjugated polyene chain composed of alternating double and single carbon-carbon bonds that are responsible for their photochemical and antioxidant activities (115, 116). Carotenoids are absorbed similarly to lipids, with absorption influenced by dietary cholesterol intake (117).

The association between high doses of *β*-carotene, lutein, and zeaxanthin supplementation and the rate of NMSC has been inconsistent at best, with many clinical studies finding no reduction in the incidence of cutaneous keratinocyte cancers (118–121). However, studies do support an inverse relationship between dietary carotenoid intake and overall cancer risk (122, 123). The incidence of NMSC was found to be inversely related to blood levels of *β*-carotene, while a higher dietary intake of lutein and zeaxanthin was associated with a decreased incidence of SCC in the context of secondary prevention (120, 124). Although carotenoid supplementation was not found to offer any benefit against NMSCs, increased consumption of a variety of different carotenoid-rich foods might confer a degree of protection (122, 123). Retinol supplementation was independently associated with a reduced risk of melanoma (125). Although there is promise among pre-clinical studies, the small sample sizes and relatively narrow questions limit any significant conclusions that can be drawn.

## 7 Discussion

Given the elevated morbidity and mortality from skin cancer in SOTRs, effective strategies for cutaneous chemoprevention are essential to improve outcomes within this vulnerable patient population. With the current focus on surveillance and sun protection, dietary interventions are an underutilized approach with significant potential to reduce the incidence and metastatic potential of SCCs and BCCs in transplant recipients.

Taken together, the evidence suggests that dietary interventions may reduce the impact of UV-induced carcinogenesis within the context of chronic immunosuppression (Table 1). By modulating oxidative stress, enhancing DNA repair, and balancing inflammatory signaling, nutritional compounds such as omega-3 fatty acids, nicotinamide, polyphenols, and carotenoids have been associated with a reduced incidence of AKs, NMSCs, and melanoma. These compounds can likely work together in a synergistic manner, suggesting a comprehensive, integrated dietary approach may provide an effective, low-risk option for cutaneous chemoprevention.

**Table 1:** A summary table classifying the evidence included based upon the studied group (SOTR, other immunocompromised, immunocompetent, and pre-clinical) and among the clinical studies, whether the focus was on primary or secondary prevention.

Nutritional strategy	Dietary strategies	Omega-3 & Omega-6 Fatty Acids	Nicotinamide, Niacin, and other Vitamin B3 Derivatives	Phytochemicals, Polyphenols, & Carotenoids
Solid organ transplant recipients 1° prevention		Miura et al, 2019—Pilot double-blind RCT with 49 lung transplant recipients finding signal for reduced skin cancer incidence. Miura et al, 2020—In a cohort study of 449 kidney and liver transplant recipients using food frequency questionnaires, SOTRs with high long-chain omega-3 fatty acid intake demonstrated reduced SCC tumour risk, while those with high intake of the omega-3 alpha-linoleic acid specifically had lower BCC incidence.		
Solid organ transplant recipients 2° prevention			Allen et al, 2023 (ONTRANS)—Phase III double-blind RCT with 158 SOTRs finding no difference in AK and NMSC incidence at 12 months between oral nicotinamide supplementation and placebo. Chen et al, 2016—This phase II double-blind RCT with 22 kidney transplant recipients and a prior history of NMSC found a statistically nonsignificant reduction in NMSC and AKs. Breglio et al, 2025—Early nicotinamide use among 1334 SOTRs was associated with reduced SCC incidence in a retrospective cohort study using Veterans Affairs data. Zhang et al, 2023 –In an open-label RCT, nicotinamide did not decrease AK development among 21 kidney transplant recipients with a history of NMSC. Drago et al, 2017—In a case-control series with 38 liver and kidney transplant participants, oral nicotinamide at 500 mg daily was associated with reduced AK size and formation compared to controls at 6 months. Hwang et al, 2025—In a retrospective study using a pre-post cohort design of 47 SOTRs, nicotinamide supplementation was associated with reduced NMSC incidence.	
Other immunosuppressed populations 2° prevention		Gui et al, 2023—In a double-blind RCT of 63 patients with stage II-III non-small cell lung cancer undergoing postoperative chemotherapy, supplementation with omega-3 fatty acids was associated with lover levels of CRP and inflammatory cytokines.		
Immunocompetent populations 1° prevention	Leone et al, 2020—Nested case-control with 505 participants finding those with better adherence to the Mediterranean and DASH diet had reduced incidence of BCC. No association found between a pro-vegetarian dietary pattern and BCC. Gamba et al, 2013—*post-hoc* analysis of Women's Health Initiative Dietary Modification RCT finding a low-fat diet (fat intake 20% or less of total calories) among 48, 835 postmenopausal did not reduce incidence of NMSC. van Dam et al, 2000—Analysis of Health Professionals Follow-up Study using semiquantitative food-frequency questionnaires in men finding very minor associations between higher monounsaturated fat, higher alpha-carotene, and lower folate intake with BCC risk. Mahamat-Saleh et al, 2019—Analysis of the Etude Epidémiologique auprès de femmes de la Mutuelle-Générale de l’Éducation Nationale using food questionnaires finding among a cohort of 67332 women, better adherence to the Mediterranean Diet was associated with a lower risk of skin cancer, notably melanoma and BCC. Wei et al, 2025—Cross-sectional analysis using 45409 participants from NHANES finding no overall association between dietary inflammatory index and skin cancer prevalence.	Hughes et al, 2009—Community-based cohort study of 1119 residents finding decreased AK acquisition among the highest consumers of oily fish and wine. No consistent association between dietary patterns and AK. Pilkington et al, 2013—Double-blind RCT of 79 women with nickel allergies finding participants taking 5 g of an omega-3 polyunsaturated fat supplement had less photoimmunosuppression compared to participants taking the control lipid. Pilkington et al, 2014—Double-blind RCT of 79 women with nickel allergies finding an augmented eicosapentaenoic acid to arachidonic acid ratio among participants taking the omega-3 supplement.	Adler et al, 2026—Retrospective cohort study using TriNetX of 991872 participants finding an association between niacinamide exposure and reduced 10-year incidence of NMSC and melanoma.	Kune et al, 1992—In a case-control study of 176 participants, a lower intake of beta-carotene and vitamin C-containing foods was associated with higher NMSC incidence. Asgari et al, 2012—Using 69635 participants from the VITamins And Lifestyle (VITAL) cohort, retinol supplementation was associated with a reduction in melanoma risk, but there were no associations between melanoma risk with vitamin A or carotenoid intake. Kim et al, 2019—Cohort study using 123570 participants from the Nurses’ Health Study and the Health Professionals Follow-up Study finding higher total vitamin A intake, retinol, and some carotenoids were associated with a reduction in SCC risk. Song et al, 2012—In a cohort analysis using 112897 participants from the Nurses’ Health Study and the Health Professionals Follow-up Study, there was an inverse association between caffeine intake from all sources and BCC risk was found. Sui et al, 2024—Umbrella meta-analysis of 198 meta-analyses finding increased dietary intake and blood concentrations of carotenoids are significantly association with a reduced total risk of cancer. Ojobor et al, 2023—In a systematic review and meta-analysis of 198 observational studies and 50 prospective studies, increased carrot intake and higher alpha-carotene levels was significantly association with an overall decrease in cancer risk. Farrar et al, 2015—Double-blind RCT of 50 participants found green tea extract with vitamin C does not significantly reduce skin erythema, leukocyte infiltration, or eicosanoid response to UVR. Moehrle et al, 2008—Among 15 male physician participants, oral or topical use of red wine in general does not impact minimal erythema dose, but there may be higher sun tolerance with wine that has a high polyphenol content.
Immunocompetent populations 2° prevention	Black et al, 1995 – RCT with 101 NMSC patients finding a significant reduction in skin cancer incidence among the low fat (20% or less) group. Ibiebele et al, 2009 – Longitudinal food-based questionnaire with 1057 participants finding no overall trend existed, but increased total fat intake was associated with more SCCs and no associations with BCC incidence. Malagoli et al, 2015 – In a case-control study using a semiquantitative food frequency questionnaire among 1099 participants with a prior diagnosis of non-metastatic melanoma, there was an inverse association between melanoma risk with the Health Eating Index 2010 and DASH diets, but not with Mediterranean diets.	Hakim et al, 2009 – Case-control study of 568 participants from the Southeastern Arizona Skin Cancer Registry with prior non-metastatic SCC finding decreased risk of SCC among diets with higher omega-3 to omega-6 ratios.	Chen et al, 2015 (ONTRAC) – A phase III double-blind RCT with 386 participants with prior NMSC finding a significant reduction of AKs and NMSC in the group receiving 500 mg BID nicotinamide supplementation over a 12 month period. Breglio et al, 2025 – Retrospective cohort study using Veterans Affairs Corporate Data Warehouse of 33 822 patients who were predominantly immunocompetent finding a significant reduction in NMSC risk with oral nicotinamide supplementation, especially when initiated earlier on for 2° prevention. Surjana et al, 2012 – Phase I double-blind RCT with 74 participants who had prior AKs finding a reduction in AK count with daily 500 mg nicotinamide supplementation.	Heinen et al, 2007 – Prospective cohort study of 1001 participants using semi-quantitative questionnaires every 6 months finding higher dietary intake of lutein and zeaxanthin was associated with reduced SCC incidence for 2° prevention while vitamin C and E in participants without a history of skin cancer was associated with the development of BCC. Fung et al, 2002 – Analysis of 5392 cases from the Nurses’ Health Study using semiquantitative food-frequency questionnaires finding no evidence that vitamins A, C, E, folate, or carotenoids play a protective role against the incidence of SCC.
Mechanistic and pre-clinical evidence		Lou et al, 2011 – Mice fed a high-fat fish oil diet rich in omega-3s had increased latency and small tumour formation compared to those fed a high-fat mixed-lipids diet rich in omega-6. Fischer et Black, 1991 – UV-radiated mice fed omega-3 fatty acids demonstrated a reduced response to inflammatory stimuli compared to omega-6 fed mice. De Caterina et al, 1995 – Cellular *in vitro* incorporation of the omega-3 fatty acid docosahexaenoic acid into cell membranes decrease the production of pro-inflammatory cytokines. Kudryavtsev et al, 2005 – *In vitro* study finding that metabolites of the omega-6 polyunsaturated fatty acid arachidonic acid is a growth-limiting factor for tumour cells.	Gensler et al, 1999 – A dose-dependent preventative effect on photocarcinogenesis was found in cells derived from UV-irradiated mice given different levels of niacin supplementation. Park et al, 2010 – Nicotinamide prevented UV-induced cellular ATP loss and glycolytic blockade in keratinocytes.	Zhao et al, 1999 – In hairless mice and skin from human volunteers, topical and oral administration of black tea extract was found to reduce the degree of erythema and skin thickness following UVB exposure. Caltagirone et al, 2000 – Administration of the flavonoids apigenin and quercetin inhibit the growth and metastatic potential of melanoma cells in mice. Mnich et al, 2009 – Green tea extract was found to reduce induction of p53 and apoptosis in UVB-irradiated human skin. Matito et al, 2011 – *In vitro* study finding protective effects against UV-induced oxidative damage with grape polyphenols. Nihal et al, 2004 – The main polyphenol in green tea, EGCG, can decrease the viability and growth of melanoma cell lines.
Current recommendations	The current literature suggests there is limited or weak evidence that the Mediterranean and DASH diets may help for primary prevention of NMSC, and potentially some exploratory evidence of secondary prevention with low-fat diets, especially when considering the data around omega-3 fatty acids. These diets could potentially be recommended to SOTRs.	Evidence from SOTRs, other immunosuppressed populations, immunocompetent populations, and mechanistic studies suggest possible benefit from omega-3 fatty acids for the primary and secondary prevention of NMSC. Based on the existing evidence, increasing intake of omega-3 polyunsaturated fatty acids should be recommended to SOTRs.	Conflicting evidence, with RCTs generally finding no statistical benefit (although many were underpowered compared to the calculated samples in power analyses) with potential associations in observational studies. A signal possibly exists for primary prevention with nicotinamide supplementation for SOTRs and could be a conditional recommendation.	Studies are predominantly exploratory, mechanistic, or observational. Further investigations are needed before any recommendations can be provided.

Despite the biologic plausibility and emerging clinical signals, significant gaps remain. Most evidence is derived from immunocompetent populations or preclinical models, and transplant-specific trials have been limited by small sample sizes, short follow-up, and challenges with adherence. However, the absence of consistent high-quality evidence does not negate the potential benefit these nutrients hold, especially given their exceedingly low risk when increased through direct dietary modification. Future clinical trials with SOTRs should prioritize pragmatic, well-powered studies that focus on overall dietary patterns rather than isolated nutrients.

From a clinical perspective, these considerations support a shift towards the integration of dietary counseling within standard transplant dermatology care. Emphasis should be placed on dietary patterns rich in plant-based foods and omega-3 fatty acids while avoiding foods with excessive amounts of omega-6 fatty acids, while still noting they are essential for a healthy diet. The focus should be on well-established approaches, such as the Mediterranean diet, or the nutritional recommendations provided in cancer risk reduction guidelines. Selective use of supplementation, like omega-3 fatty acids or nicotinamide, may be considered, especially in the context of primary chemoprevention. Multidisciplinary teams involving dieticians, social workers, and other specialists should be involved when providing dietary recommendations, and equitable and affordable access must be treated as a priority, especially in low-income and low-access regions. Importantly, such strategies should complement rather than replace established interventions like photoprotection and pharmacologic chemoprevention through systemic retinoids, and monitoring should be done to look for any potential nutrient-drug interactions.

Rather than being treated as a peripheral lifestyle factor, we argue that diet must be viewed as a modifiable determinant of cutaneous carcinogenic risk that plays an essential role in determining long-term outcomes for SOTRs. Given the substantial and persistent burden of skin cancer in this population, dietary education should be utilized as a potentially effective, low-risk approach to ensure transplant recipients can live longer, more fulfilling lives.

## Data Availability

The original contributions presented in the study are included in the article/Supplementary Material, further inquiries can be directed to the corresponding author.
